# Searching for master regulators of transcription in a human gene expression data set

**DOI:** 10.1186/1753-6561-1-s1-s81

**Published:** 2007-12-18

**Authors:** Alfonso Buil, Alexandre Perera-Lluna, Ramon Souto, Juan M Peralta, Laura Almasy, Montserrat Vallverdu, Pere Caminal, Jose M Soria

**Affiliations:** 1 Unitat de Bioinformatica i Malalties Complexes, Institut de Recerca de l'Hospital de la Santa Creu i Sant Pau, Barcelona, 08025 Spain; 2 Centre de Recerca en Enginyeria Biomedica, Universitat Politecnica de Catalunya, Barcelona, 08034 Spain; 3 Department of Genetics, Southwest Foundation for Biomedical Research, San Antonio, Texas 78245, USA

## Abstract

Microarray technologies allow the measurement of the expression levels of thousands of transcripts at the same time. As part of Genetic Analysis Workshop 15 (GAW15), we analyzed a data set that measured the expression of more than 3000 genes in 14 families. Our goal was to identify genomic regions that regulate the expression of several genes at the same time. We tried two different approaches: one was maximum likelihood-based variance-component linkage analysis and the other was a new linkage regression approach. We detected some loci that were linked with the expression level of more genes than would be expected by chance. These loci are candidates for master regulators of transcription (MRT). Finally, for each candidate MRT, we did a gene ontology (GO) analysis to test whether the genes linked to it were biologically related.

## Background

One common feature observed in studies of transcript expression is the presence of hot spots, that is, individual loci that affect large number of transcripts [1]. Hot spots are defined as those loci for which the number of linked (or associated) transcripts exceeds that expected if loci influencing transcripts were randomly distributed along the genetic map. Some authors call these hot spots "master regulators of transcription" (MRT) [2], implying that an observed hot spot is due to a common quantitative trait locus (QTL) that regulates several transcripts simultaneously.

In this study we applied two different linkage strategies to localize hot spots of transcript expression in the Genetic Analysis Workshop 15 (GAW15) Problem 1 data. On the one hand, we used the maximum likelihood-based variance components linkage method and on the other, we tried a new linkage regression approach that implements a feature selection algorithm. Finally, we tried to identify commonalities among the different genes that seem to be regulated by each MRT using the information given by the gene ontologies (GO) [3].

## Methods

### Data

We used the Problem 1 data of GAW15 as described by Mosley et al. [2]. These data consisted of 196 individuals from 14 CEPH (Centre d'Etude du Polymorphisme Humain) Utah families with seven to eight offspring per family. For each individual, we have the expression levels of 3554 transcripts (the phenotypes) and 2882 autosomal and X-linked single-nucleotide polymorphisms (SNPs) (the genotypes). See the GAW15 data description for more information [4].

### Pre-processing

Mendelian errors and double-recombinants were blanked according to mistyping probabilities estimated by SIMWALK2 [5] using its default options. We blanked 119 genotypes as Mendelian errors and 570 genotypes as double-recombinants. Genetic map positions were obtained using the SNP Mapping web application developed at the University College Dublin Conway Institute of Biomolecular and Biomedical Research, located at . We estimated multipoint identity-by-descent (IBD) matrices using all of the genotyped SNPs with Merlin [6]. Given that linkage disequilibrium (LD) between pairs of SNPs was low (*r*^2 ^< 0.2), we ignored it when creating the IBD matrices.

### Univariate linkage analysis by variance components

We performed variance components linkage analysis with SOLAR [7] for each of the 3554 phenotypes. We fitted models with additive genetic, QTL, and residual environmental variance components and we used sex as a covariate. We characterized a hot spot as a locus with more than four phenotypes having LOD scores greater than 3.4.

### Linkage by feature selection regression

We present a regression method based on the Haseman-Elston approach [8]. Given a phenotype, the mean-corrected product of the sibs' trait values [(*x*_1_-μ) (*x*_2_-μ)] was used as a measure of phenotypic distance and the IBD estimates between pairs were used as measures of the genetic similarity at each genetic location. In the standard Haseman-Elston regression, the phenotypic distance is regressed on the genetic similarity, for a set of locations along the genome (usually for every centimorgan). The result is a linkage test for each location. Here we tried a different approach: given a phenotype we tried to select the set of genetic locations that best explained the phenotypic distances. Instead of models that test linkage at only one location, we allowed models that combined locations. The linkage analysis result for a phenotype is the combination of genetic locations that best explains the phenotypic distances between sib pairs.

The selection of genetic locations related to a specific phenotype based on exhaustive search is computationally impractical with current computer capabilities: the number of combinations rises up to 2^*n *^where *n *is the number of locations. In these cases, sub-optimal algorithms can be used to find a selection. This sub-optimality presents the risk of falling to local minima in the solution.

The sequential backward selection (SBS) algorithm [9] begins with a model that contains the whole set of *n *features as the initial subset, and applies the following steps: 1) evaluate a quality criterion for the current model; 2) try dropping each of the features in the subset and compute the corresponding criterion; 3) select the best candidate model; 4) iterate until the quality criterion is not improved.

The number of combinations needed for this solution will obviously be less than for an exhaustive search. In the symmetric algorithm, namely sequential forward selection (SFS), the initial subset contains zero elements and step two (above) includes a feature instead of dropping it.

Note that when one feature is dropped in SBS, there is no possibility of including it again in the subset, although it could provide further information and improve the criterion. To avoid this nesting effect, a variant family of the above algorithms called "floating search methods" has been proposed [10]. For sequential forward-floating selection (SFFS) the algorithm begins with an empty feature set. At each step, the best feature that satisfies the quality criterion is included in the current data set (SFS step). If the criterion is improved by removing some of the features in the new data set, the algorithm performs a backward step (SBS). Therefore, SFFS dynamically increases and decreases the number of features until no improvement is found with any step.

A SFFS algorithm has been built for determining genetic locations related to a phenotype. The floating search algorithm targeted the minimization of the root mean square error (RMSE) of a partial least squares (PLS) model when predicting a given phenotypic distance from the complete set of loci. For each model constructed, the number of PLS optimal latent variables was obtained from the training set (one-third of the available pairs) by means of four-fold cross-validation. RMSE was computed on the validation set, covering two-thirds of the available pairs.

We applied this algorithm to the 3554 phenotypes. Because the SFFS algorithm is computationally intensive, instead of using all the available genetic locations (one for every centimorgan), we used only 800 locations evenly spaced, one every 5 cM. Results are given as a vector of the locations (expressed in cM) selected for each phenotype.

### Gene ontology analysis

We performed tests to evaluate statistical over-representation of gene ontology (GO) [3] categories in sets of genes controlled by the same hot spot, using the Biological Network Gene Ontology (BiNGO) tool [11]. This tool performs a hypergeometric test with a Benjamini & Hochberg false-discovery rate (FDR) multiple testing correction against each of the ontologies: biological process, molecular function, and cellular component. We used a significance threshold of 0.01.

## Results

### Variance components

We followed a classical variance-components approach and we found 11 candidate MRT (hot spots), see Table 1. Two of them have been described by Morley et al. [2] but we found 9 new ones. This suggests that the variance components approach, using the information from the grandparents, allows the identification of more MRT than the Haseman-Elston regression approach without grandparents as used by Morley et al. [2].

**Table 1:** Master Regulators of Transcription (MRT)

Chr	cM	GO-ID	GO-Description	Regression
3	24	278	M phase of mitotic cell cycle	Yes
		50684	regulation of mRNA processing	
4	65	16740	Transferase activity	Yes
4	95	16932	Transferase activity, transferring glycosyl groups	
6	44	30333	Antigen processing	
6		45012	MHC class II receptor activity	
7	142	5353	Fructose transporter activity	Yes
7	86	4324	Ferredoxin-NADP+ reductase activity	
13	108	8240	Tripeptidyl-peptidase activity	Yes
14	93	16070	RNA metabolism	Yes
14		51252	Regulation of RNA metabolism	
14		43283	Biopolymer metabolism	
14		166	Nucleotide binding	
20	54	4645	Phosphorylase activity	
6	95	--	--	
11	140	--	--	

The table shows the hot spots we found with the variance components method and the Gene Ontology (GO) over-represented terms for each of them. The last column indicates which hot spots were replicated with the feature selection regression method.

### GO analysis

We performed a GO analysis of the groups of genes controlled by the same MRT. Over-representation of GO terms in 9 of the 11 sets of genes was found at a significance level of 0.01, either in the biological process or in the molecular function ontologies (Table 1). This result suggests that these groups of genes are biologically related. We propose that this fact supports the idea that some of the candidate MRT (hot spots) are real MRT.

### SFFS algorithm

We tried a new linkage method by regression with floating variable selection. This approach allowed us to test linkage models with combinations of genetic locations. In principle, this strategy could use the maximum likelihood approach, but with such a huge data set as that of GAW15, it is computationally unfeasible. Regression linkage methods are faster than maximum likelihood-based linkage methods, which allows one to try complicated strategies in large data sets. However, our method had some drawbacks. We observed that for most of the phenotypes the regression method selected a model with just one locus. This fact may reflect the underlying reality that there is only a major gene controlling the expression of each phenotype or, more likely, it reflects lack of power because we are dealing with a small sample. On the other hand, the regression method resulted in 17 hot spots; 5 of them also were found with the variance-component approach (Table 1) and presented over-representation of GO terms. However, 12 of them are new and the GO analysis for these new hot spots did not show groups of biologically related genes. This suggests that this approach may be giving too many false positives. Therefore, a careful evaluation of the SFFS method is needed before concluding that the results are truly representative.

## Conclusion

We found 11 candidate MRT using the variance-components linkage method and 17 candidate MRT using the new regression with feature selection linkage method. Five of these MRT were found using both methods and moreover showed GO over-represented terms. Thus, these 5 are our best candidates for the real MRT. However, we want to emphasize that it is very difficult to be certain that those hot spots are true MRT [12]. Although the GO analysis can give some information, these results are not conclusive. More analyses are needed before a firm conclusion about MRT loci can be drawn.

## Competing interests

The author(s) declare that they have no competing interests.
